# 19. THE IMPLEMENTATION OF COGNITIVE ADAPTATION TRAINING (CAT) ACROSS COUNTRIES

**DOI:** 10.1093/schbul/sby014.074

**Published:** 2018-04-01

**Authors:** Dawn Velligan

**Affiliations:** University of Texas Health Science Center at San Antonio, School of Medicine

## Abstract

**Overall Abstract:** Cognitive Adaptation Training is a psychosocial treatment using environmental supports such as signs, checklists, electronic devices and the organization of belongings to cue and sequence adaptive behavior in the home or work environment. In randomized trials, CAT has been found to improve multiple domains of functional outcome for individuals with schizophrenia including, social and occupational functioning, recidivism, medication follow through and community adjustment. Implementation of CAT has taken multiple forms across various countries. In Canada, a model has been adopted in which CAT is provided by occupational therapists (OT) for 4 months. During this time. the OT trains a caseworker to continue CAT after the 4 month time frame. This method has produced large effect sizes for sustained functional changes over time in patients receiving CAT treatment. In the Texas Money Follows the Person Behavioral Health Pilot, CAT has been used in conjunction with substance use treatment and managed care service coordination to assist people in moving from nursing facilities to community living. 65% of all enrollees in the program have sustained independent living at 1 year. Improvements in functional outcome are evidenced by significant improvement in social and occupational role functioning, community living and quality of life. In addition, cost savings to the state for these Medicaid recipients is sizeable. It takes only 1.4 additional months of community residence to recover intervention costs; 97% of participants met these criteria. Also in Texas, in a program for high utilizers of hospital and emergency services, CAT provided by bachelor’s and master’s level psychologists has been used to identify the unique cause of multiple hospitalizations and emergency visits, set up supports to address these problems, and reduce inappropriate service utilization. This program has reduced hospitalizations by 80% and saved an average of $40,000 per participant across 9 months. In Finland, CAT has been used to reduce the need for sheltered housing and for improving the quality of life for outpatients with psychosis. CAT has been found to fit well in the Finnish service system and providing interventions in the patient’s daily living environment has been found to be more suited to the patients’ need than traditional outpatient clinic visits. An outcome study is ongoing. In Australia, CAT has been adapted for the recent onset psychosis population by including greater use of technology and focusing on return to work and school. The versatility of the CAT program, its facility for training, and its ability to be adapted across cultures and countries suggests that CAT could be a useful tool in value-based care in which CAT is used to reduce the overall cost of care and to improve outcomes that matter to patients.

